# Kaempferol restores the susceptibility of ESBLs *Escherichia coli* to Ceftiofur

**DOI:** 10.3389/fmicb.2024.1474919

**Published:** 2024-12-11

**Authors:** Peng-Cheng Li, Yin-Chao Tong, Xing-Lan Xiao, Yun-Peng Fan, Wu-Ren Ma, Ying-Qiu Liu, Shen Zhuang, Su-Zhu Qing, Wei-Min Zhang

**Affiliations:** ^1^College of Veterinary Medicine, Northwest A&F University, Yangling, China; ^2^Institute of Traditional Chinese Veterinary Medicine, Northwest A&F University, Yangling, China

**Keywords:** ESBLs *Escherichia coli*, kaempferol, ceftiofur, biofilm, antibiotics adjuvant

## Abstract

**Introduction:**

The development of extended-spectrum-beta-lactamase (ESBLs) *Escherichia coli* (*E. coli*) has become a global threat to public health. An alternative strategy to alleviate this is identifying potential natural compounds to restore antibiotic activity against ESBLs *E. coli*. This study aimed to find a possible compound to restore ESBLs *E. coli* sensitivity to ceftiofur.

**Methods:**

The synergistic effect of kaempferol and ceftiofur against ESBLs *E. coli* was investigated by checkerboard assays, time-kill, growth curves, and scanning electronic microscope. The impact of kaempferol with ceftiofur on the biofilm of ESBLs *E. coli* was evaluated by crystal violet staining and laser scanning confocal microscopy and this study also assessed the effect of kaempferol on the initial adhesion and aggregation of *E. coli* (SY20) by examining motility, adhesion, and surface characteristics. The RT-qPCR was used to determine the effect of kaempferol on the expression of genes related to the *LuxS*/AI-2 quorum sensing system in ESBLs *E. coli*, and the effect of kaempferol on AI-2 signaling molecules was determined by molecular docking and bioassay. The impact of kaempferol on the activity of *bla_CTX-M-27_* protein was determined by RT-qPCR, molecular docking, and nitrofen experiments, the results were further verified by transcriptome analysis. The mouse infection model was established, and the inhibitory mechanism of kaempferol with ceftiofur on bacteria in vivo was further verified by HE staining and immunohistochemistry.

**Results and discussion:**

Kaempferol with ceftiofur exerts synergistic antibacterial and bactericidal effects on ESBLs *E. coli* by influencing β-lactamase activity, biofilm formation, and *LuxS*/AI-2 QS system. In vivo, kaempferol protected the small intestinal villi from the damage of ESBLs *E. coli*. Furthermore, kaempferol fully restores the activity of ceftiofur in animal infection models by relieving the TLR_4_/NF-κb pathway. In conclusion, the sensitivity of ESBLs *E. coli* to ceftiofur in vitro and in vivo could be enhanced by kaempferol, which showed that kaempferol may be a kind of antibiotic adjuvant.

## 1 Introduction

Antibiotics were used to cure bacterial infections in the past (Muñoz et al., 2024). However, with the decline of antibiotic discovery and the evolution of drug resistance, current antibiotic resistance has become a public health crisis (Hutchings et al., 2019)^.^ Therefore, it is urgent to explore the mechanism of antibiotic resistance and find a way to eliminate drug resistance in bacteria (Aleksandrowicz et al., 2023).

It is found that certain combinations of Chinese herbal extracts and antibiotics exhibit synergistic effects against *E. coli* through distinct mechanisms (Porras et al., 2021)^.^ For instance, Quercetin could let *E. coli* regain susceptibility to colistin by enhancing its destructive effects by destroying the cell membrane of *E. coli* (Lin et al., 2021). Artesunate enhanced the inhibitory effect of various *β*-lactam antibiotics against MDR *E. coli* by inhibiting the expression of efflux pump genes (Wei et al., 2020). Magnolol enhanced the sensitivity of MDR *E. coli* to cefquinome and reversed the resistance of MDR *E. coli* (Tong et al., 2023b).

Kaempferol belongs to flavonoids, which can be found in a variety of herbs. Besides anticarcinogenic and anti-inflammatory effects, kaempferol and its extensions also show antibacterial, antifungal, and antiprotozoal effects (Periferakis et al., 2022). In addition, kaempferol has excellent anti-diabetic effects (Yang et al., 2022) and neuroprotective effects (Chang et al., 2022). The previous research showed that some kaempferol derivatives had inhibitory effects on *E. coli* biofilm (Periferakis et al., 2022)^,^ and they could also destroy the integrity of bacterial cell membranes (Lin et al., 2020).

This study aimed to explore the mechanism of kaempferol restoring the sensitivity of ESBLs *E. coli* to ceftiofur, focusing on the biofilm formation and *β*-lactamase, and the therapeutic effect *in vivo* was also studied.

## 2 Results

### 2.1 Ceftiofur and kaempferol susceptibility testing

To explore the antimicrobial activity of Ceftiofur and Kaempferol, the Minimal Inhibitory Concentration (MIC) of 6 ESBLs *E. coli* isolates and ATCC®25922™ were detected. The result showed that all 6 ESBLs *E. coli* isolates were resistant to ceftiofur (Table 1).

**Table 1:** MIC values and FICI values of Ceftiofur and kaempferol to ATCC^®^ 25922^TM^ and 6 ESBLs *E. coli* isolates.

Isolates	Ceftiofur MIC values/μg/mL	Kaempferol MIC values/μg/mL	FICI values
ATCC25922	0.03125	512	0.750
SY13	4096	1024	0.188
SY20	4096	2048	0.250
SY22	512	2048	0.250
YA1-3	8192	2048	0.375
YL2	2048	1024	0.266
YL6	4096	1024	0.625

### 2.2 Synergistic of Kaempferol with ceftiofur against ESBLs *Escherichia coli*

**Figure 1:**
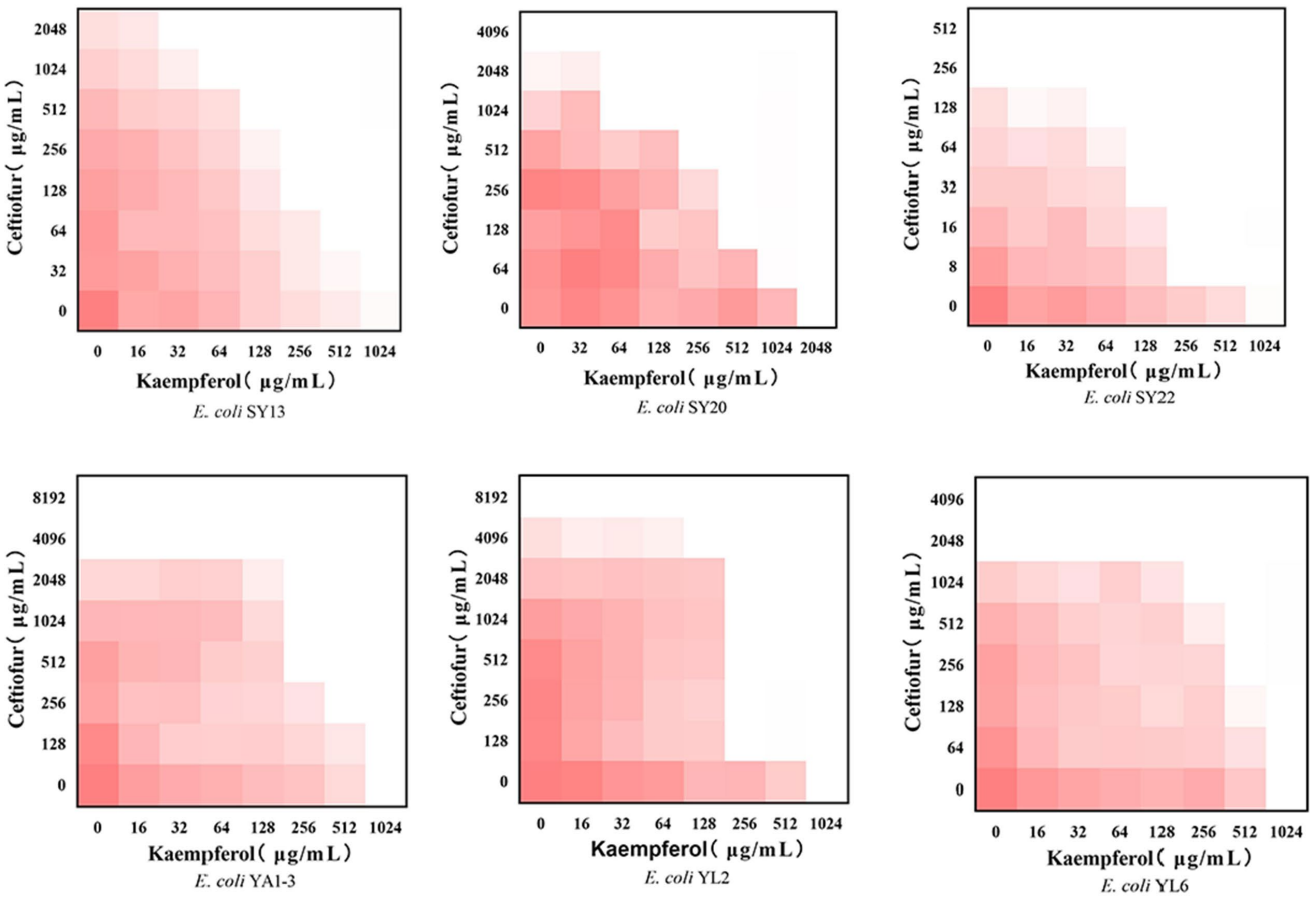
Checkerboard broth assays for kaempferol and ceftiofur against 6 ESBLs *Escherichia coli* strains. Dark red regions represent higher bacterial cell density.

To evaluate the potential synergistic effect of kaempferol with ceftiofur against ESBLs *E. coli*, checkerboard assays were performed. As shown in Table 1 and Figure 1, the rate of synergistic effects was 83.33%. The rate of additive effects was 16.67%. Notably, the FICI value of ATCC®25922™ ≤ 0.75, indicating that kaempferol combined with ceftiofur inhibited ESBLs *E coli* partially synergistically. Compared with monotherapy, the dose of ceftiofur in combination treatment was 4 to 64 lower, which suggested that kaempferol eliminated the resistance of ESBLs *E. coli* to ceftiofur.

### 2.3 Kaempferol enhances ceftiofur efficacy and minimize the emergence of resistance

Although the checkerboard test showed kaempferol to enhance ceftiofur, direct tests of synergistic inhibitory effects and synergistic bactericidal activity may reinforce these findings. Based on the results of MIC and FIC assays, three strains of ESBLs *E. coli* (SY13, SY20, SY22) were choosed for the next test.

**Figure 2:**
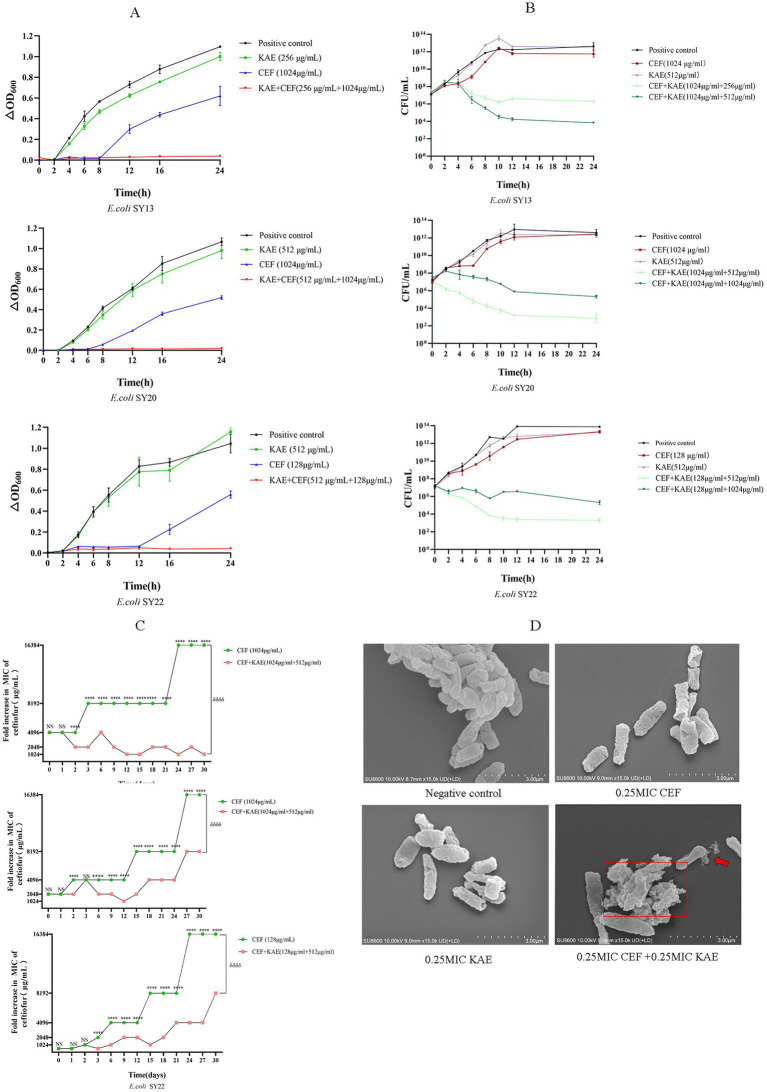
Kaempferol Enhances Ceftiofur Efficacy and Minimize the Emergence of Resistanc. (A). The growth curves. (B). Time-dependent killing. (C). The addition of kaempferol (0.25MIC) prevents the evolution of ceftiofur resistance to *E. coli*. (D). Morphological changes of ESBLs *E. coli* (SY20) with different treatments under SEM.

The growth curves were performed to analyze the inhibitory effects of the combination of kaempferol and ceftiofur against ESBLs *E. coli*. As shown in Figure 2A, compared with the CEF group and the KAE group, the CEF + KAE group showed better effects on inhibiting the growth of ESBLs *E. coli* within 2 to 24 h. The results suggested that the combination of kaempferol and ceftiofur may be an ideal bacterial-inhibiting combination.

The time-kill curves were performed to evaluate the bactericidal effect of the combination of kaempferol and ceftiofur against ESBLs *E. coli*. As shown in Figure 2B, compared with the KAE group and the CEF group, at all concentrations tested, the combination of kaempferol and ceftiofur exhibited an enhanced bactericidal effect against the three tested ESBLs *E. coli* strains within 24 h. After 12 h treatment, the differences in the total bacteria counts between the KAE + CEF group (the low concentration group) and the CEF group reached 10^5^- to 10^7^-fold, and this gap lasted for 12 h. Notably, the differences in the population between the low-concentration KAE + CEF group and the high-concentration KAE + CEF group reached 10^2^- to 10^3^-fold from 8 to 24 h, which indicated that the bactericidal effect appeared to be kaempferol dose-dependent.

Scanning Electron Microscopy (SEM) was used to observe the morphological changes of ESBLs *E. coli* (SY20) directly. As shown in Figure 2D, the surface of cells in the KAE + CEF group showed depression, shrinkage, and even rupture, and lysis, indicating that the ability of ceftiofur to disrupt cell surface structures could be enhanced by kaempferol.

Finally, to understand the role of kaempferol in the development of ceftiofur resistance, we performed serial passages of ESBLs *E. coli* with ceftiofur (0.25 MIC) in the presence and absence of kaempferol (0.25 MIC) during 30 d. As shown in Figure 2C, the growth of the ESBLs *E. coli* resistance in the KAE + CEF group was significantly lower than that in the CEF group (*p* < 0.001), this indicates that kaempferol can slow down the development of ESBLs *E. coli* resistance to ceftiofur.

In conclusion, kaempferol can enhance the antibacterial and bactericidal effects of ceftiofur and minimize the emergence of ESBLs *E. coli* Resistance to ceftiofur.

### 2.4 Kaempferol enhances the biofilm-damaging ability of ceftiofur

Considering the anti-biofilm activity of kaempferol and ESBLs *E. coli* developed ceftiofur-resistance by producing β-lactamase. We speculated that kaempferol restored bacterial sensitivity to ceftiofur by affecting biofilm formation, quorum sensing, and β-lactamase activity.

**Figure 3:**
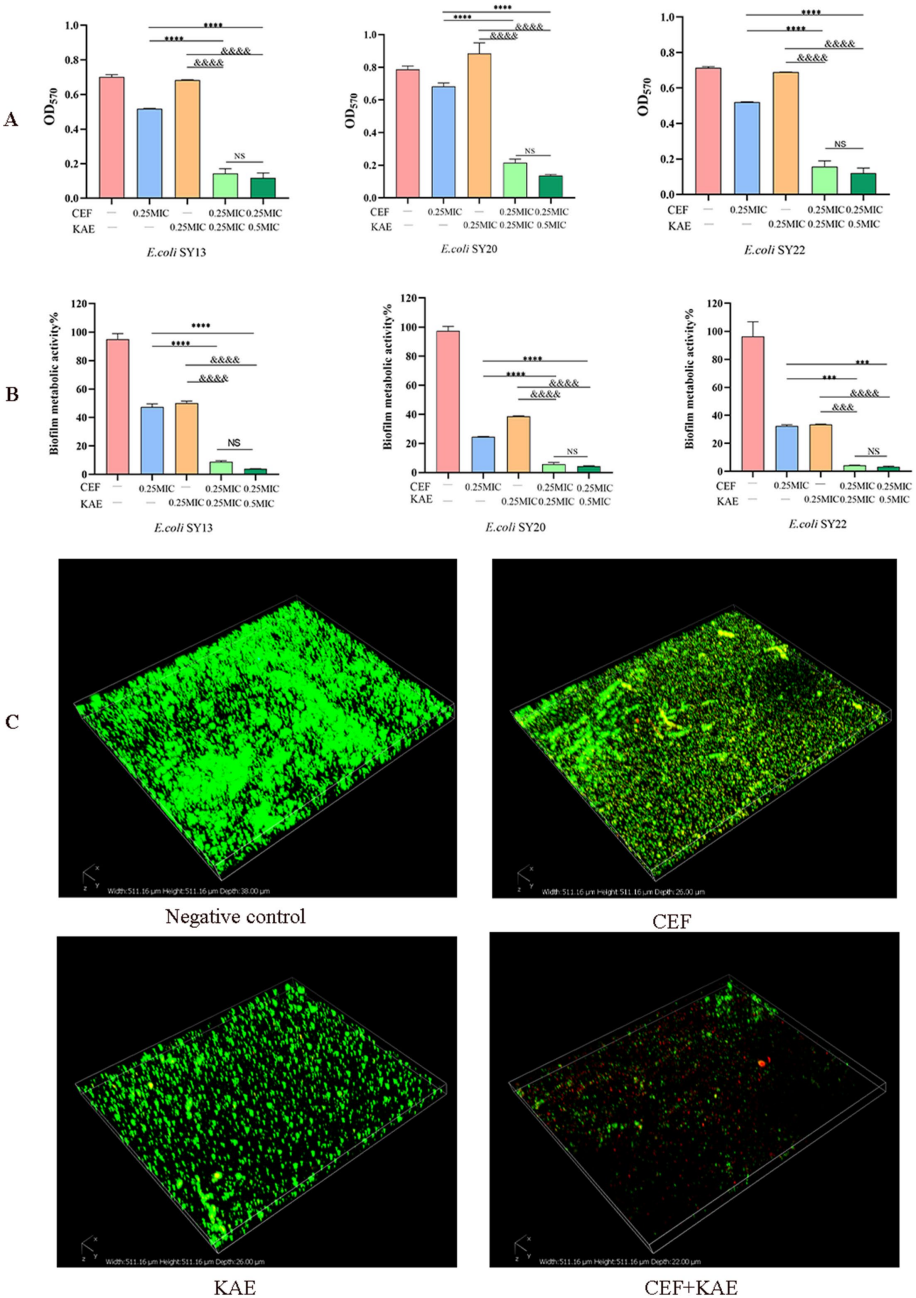
Kaempferol enhances the biofilm-damaging ability of Ceftiofur. (A) The absorbance at OD_570_ of crystal violet staining of ESBLs *E. coli* treated with Kaempferol (KAE) and Ceftiofur (CEF) in different combinations or levels. (B) The absorbance at OD_570_ of MTT staining of ESBLs *E. coli* treated with kaempferol (KAE) and ceftiofur (CEF) in different combinations or levels. (C) Biofilm of ESBLs *E. coli* (SY20) after different treatments, dead ESBLs *E. coli* in the biofilm were stained red by PI, and all the ESBLs *E. coli* were stained green by SYTO9. CEF: ceftiofur; KAE: kaempferol.

Biofilm formation of ESBLs *E. coli* was measured by crystal violet staining. As shown in Figure 3A, the absorbance of OD_570_ in the CEF + KAE group was significantly lower than in the monotherapy groups (*p* < 0.0001). The high-level kaempferol (0.5 MIC) combined with the same level of ceftiofur exhibited the same destructive effect on biofilm formation as the low-level group. The above results suggested that the combination of kaempferol and ceftiofur affects the biofilm formation in ESBLs *E. coli.*

To further investigate the effect of different treatments on the metabolic activity of biofilm cells, the Methyl thiazolyl tetrazolium (MTT) colorimetric method was performed. As shown in Figure 3B, the absorbance of OD_570_ in the CEF + KAE group was significantly (*p* < 0.0001) lower than in the monotherapy groups. The above results suggested that the combination of kaempferol and ceftiofur could affect the metabolic activity of biofilm.

To observe the destruction of different treatments on biofilm, Confocal Laser Scanning Microscopy (CLSM) was performed to evaluate the changes in biofilm activity (Figure 3C). Compared with the monotherapy group and negative control, ceftiofur combined with kaempferol killed a large population of ESBLs *E. coli* (SY20) and showed severe shedding of biofilm. Considering the phenomenon of SY20 shedding in the CEF + KAE group, we speculated that the combination of kaempferol and ceftiofur might affect *E. coli* adhesion and aggregation.

To further explore whether kaempferol affected the ESBLs *E. coli* (SY20) aggregation, we studied from three aspects: motility, adhesion, and surface characteristics.

**Figure 4:**
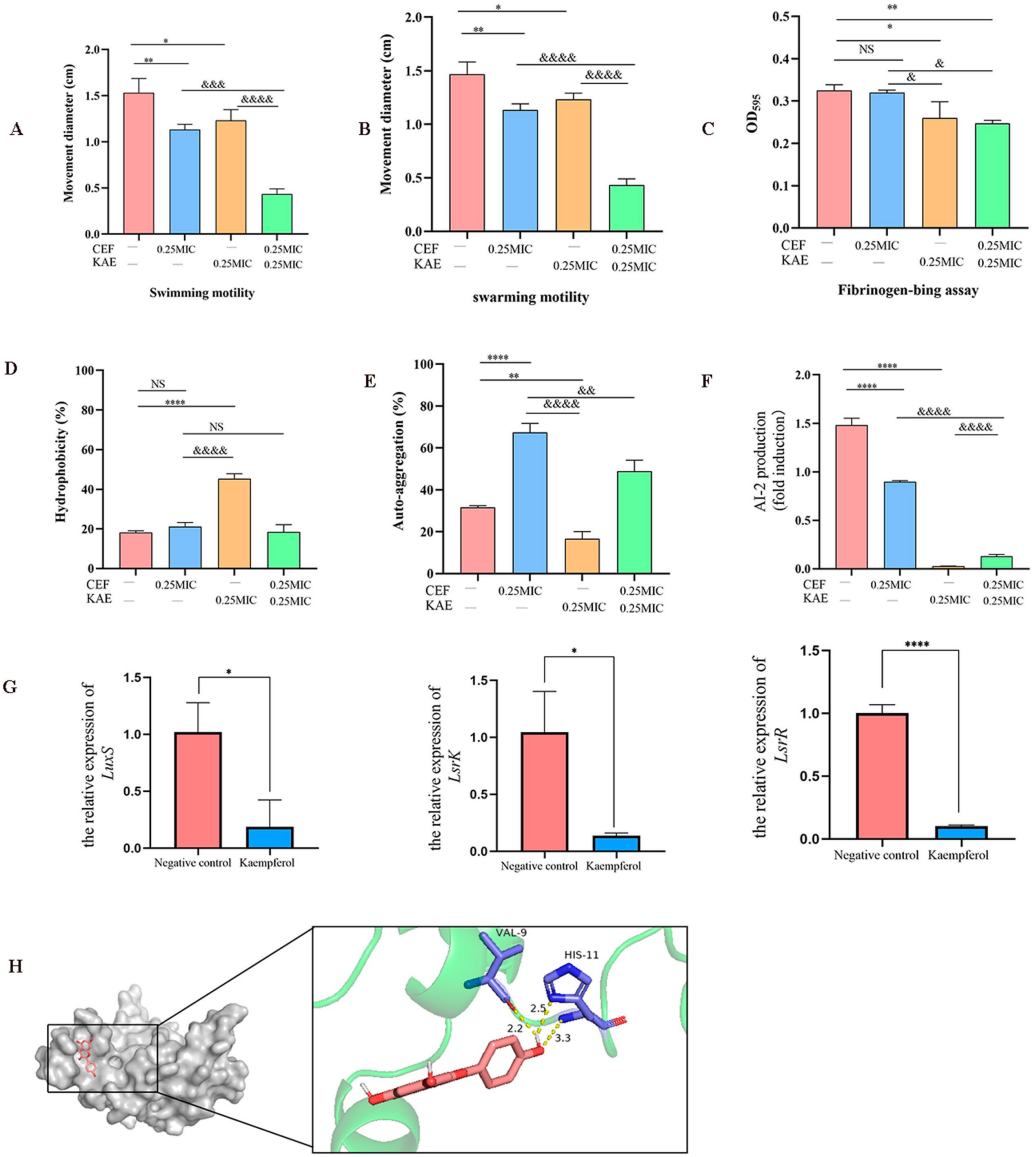
Using ESBLs *E. coli* (SY20) as an example, the effects of kaempferol on the adhesion, aggregation, and *LuxS*/AI-2 during the formation of *Escherichia coli* biofilm were measured. (A). Swimming motility. (B). Swarming.motility. (C). Fibrinogen-bing assay. (D). Hydrophobicity. (E). Self-aggregation. (F). Measurement of AI-2 activity using bioluminescence assay. (G). The relative expression of *LuxS*, *LsrR*, and *LsrK* by RT-qPCR. (H). Molecular docking of kaempferol and *LuxS* protein.

The motility of SY20 was assessed in the presence and absence of kaempferol by measuring the diameters of the swarming and swimming zones. Compared with the CEF group, the CEF + KAE group significantly prevented the swarming motility (*p* < 0.05; Figure 4A) and swimming motility (*p* < 0.001; Figure 4B) of ESBLs *E. coli*.

Then we test the effect of kaempferol on SY20 adhesion by fibrinogen-bing assay. Compared with the CEF group, the CEF + KAE group significantly reduced the adhesion ability of SY20 to the fibrinogen (*p* < 0.05; Figure 4C), the results showed that kaempferol may affect the attachment phase of biofilm formation by reducing the SY20 adhesion.

The surface characteristics (hydrophobicity and self-aggregation) of SY20 were tested to explore their relationship with aggregation in ESBLs *E. coli*. The hydrophobicity of *E. coli* can regulate their adhesion on diverse surfaces, compared with the CEF control, the KAE group significantly enhanced the hydrophobicity of SY20 (*p* < 0.0001), but there was no significant difference between this group and CEF + KAE group (*p* > 0.05; Figure 4D). The self-aggregation of *E. coli* contributes to the improvement of bacterial biofilm morphology. In the present study, we found that under the stress of ceftiofur, the self-aggregation ability of SY20 was significantly enhanced, while kaempferol could reduce this stress (Figure 4E).

On the other hand, *LuxS*/AI-2 QS as the key to regulating the biofilm formation and motility of *E. coli* were also considered. Compared with the group without kaempferol treatment, the AI-2 activities within the supernatant of biofilms of ESBLs *E. coli* with kaempferol treatment were significantly decreased (*p* < 0.001; Figure 4F). Meanwhile, the relative expression levels of the group than in the Negative control group. Finally, We found that kaempferol can bind tightly to the two residues of VAL-9 and HIS-11 in the active center of *LuxS* protein by forming three hydrogen bonds (Binding energy as −8.422 kcal/mol) (Figure 4H).

### 2.5 Kaempferol inhibits the *bla_CTX-M-27_* protein activity

Considering ESBLs *E. coli* developed ceftiofur resistance by producing *β*-lactamase, we speculated that kaempferol restored bacterial sensitivity to ceftiofur by inhibiting the β-lactamase activity.

**Figure 5:**
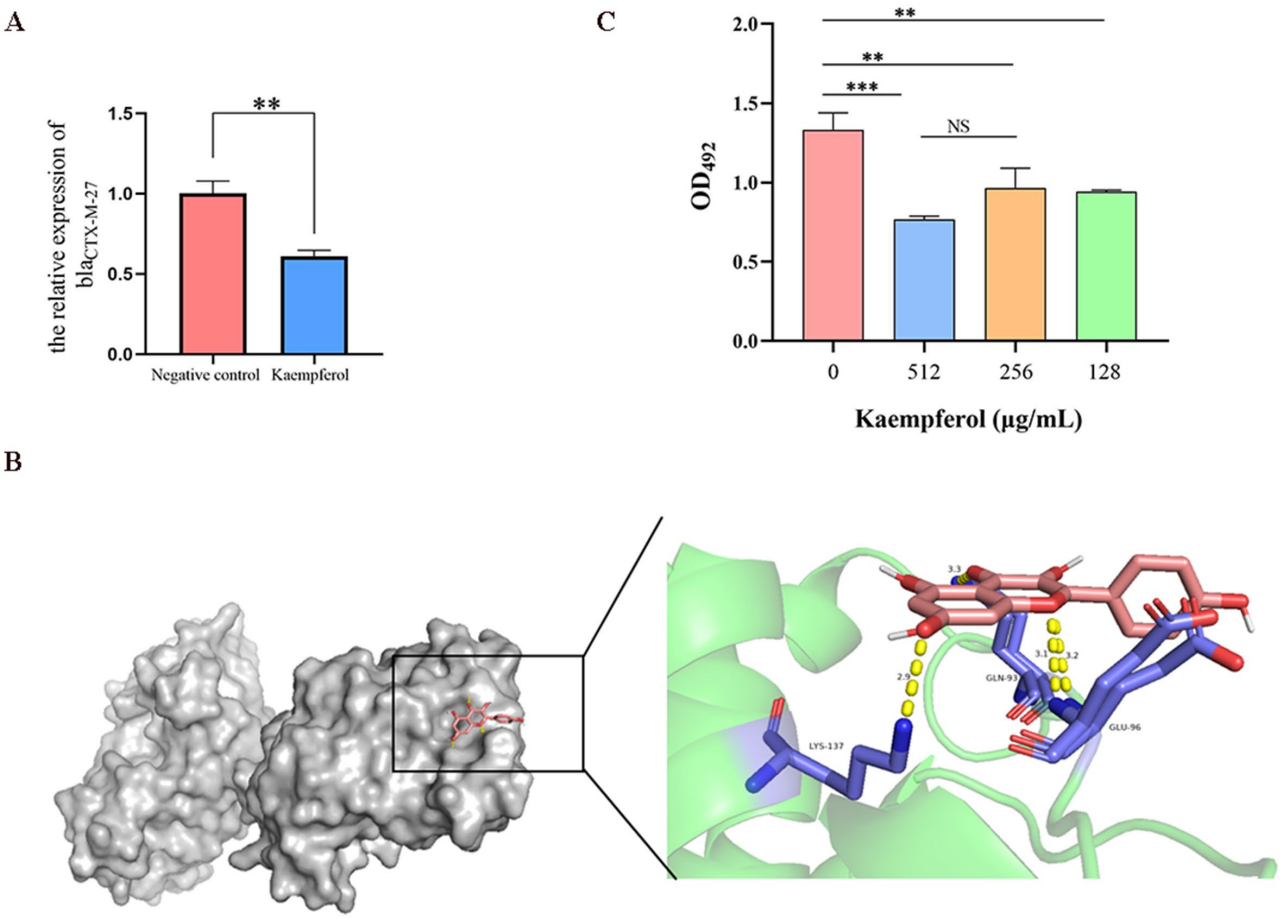
Kaempferol inhibits the *bla_CTX-M-27_* proteion activity of ESBLs *E. coli*. (A). The relative expression of *bla_CTX-M-27_* by RT-qPCR. (B). Molecular docking of kaempferol and *bla_CTX-M-27_* protein. (C). The effect of kaempferol on protein activity was determined by nitrocefin test.

RT-qPCR was used to simulate the effect of kaempferol on the expression level of the *bla_CTX-M-27_* gene and investigated the interaction between kaempferol and beta-lactamase active centers through molecular docking (Figure 5B). The result showed that kaempferol can reduce the relative expression of *bla_CTX-M-27_* and bind tightly to the active center of *bla_CTX-M-27_* (Binding energy as −8.425 kcal/mol). Therefore, nitrocefin tests were used to detect the effect of kaempferol on *bla_CTX-M-27_* protein activity. The result showed that kaempferol significantly (*p* < 0.05) inhibited the hydrolytic activity of *bla_CTX-M-27_* protein (Figure 5C). These data suggest that kaempferol has the potential to enhance cefotifo against ESBLs by inhibiting *β*-lactamase activity.

### 2.6 Transcriptome analysis was further verified

**Figure 6:**
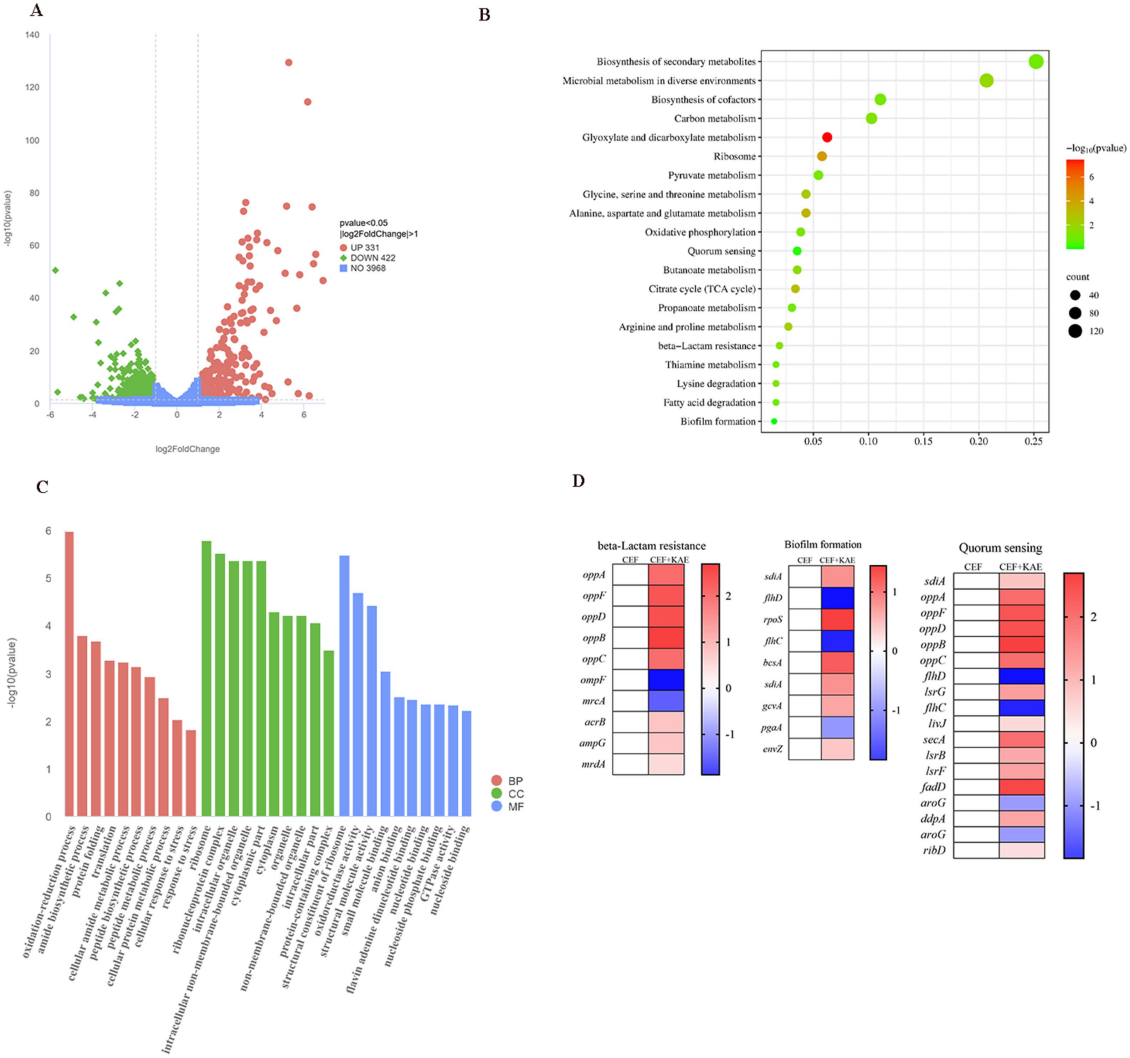
Transcriptome analysis of *E. coli* SY20 after exposure to ceftiofur alone or the combination of ceftiofur and kaempferol. (A). Volcano plot. (B). KEGG (Kyoto Encyclopedia of Genes and Genomes) enrichment analysis of the DEGs in *E. coli* SY20. (C). GO (Gene Ontology) annotation analysis of the DEGs in *E. coli* SY20. (D). Selected differential expression genes related to *E. coli* drug resistance involved in Biofilm formation, Quorum sensing system, and β-lactamase activity.

The comparison of treatment with a combination of ceftiofur alone revealed an up-regulation of 331 genes and a down-regulation of 442 genes (Figure 6A). KEGG (Figure 6B) and Go (Figure 6C) enrichment analysis showed that these differentially expressed genes (DEGs) were involved in pathways related to *E. coli* resistance, such as *β*-lactam resistance, biofilm formation, and quorum sensing, this is consistent with our previous speculation that kaempferol with ceftiofur plays a synergistic role by affecting the biofilm formation and β-lactamase activity (Figure 6D). KEGG enrichment (Figure 6B) analysis showed that KAE + CEF affected multiple metabolic pathways, including Glyoxylate and dicarboxylate metabolism, Alanine, aspartate and glutamate metabolism, Glycine, serine and threonine metabolism, Arginine and proline metabolism etc. Go enrichment analysis oxidation–reduction process, cellular protein metabolic process etc., suggests that kaempferol may restore *E. coli* sensitivity to ceftiofur by influencing *E. coli* metabolic indications, and it provides a new direction for our future research.

Collectively, transcription analyses of *E. coli* (SY20) further indicated our previous study that kaempferol enhanced the inhibitory and killing effect of ceftiofur on ESBLs *E. coli* by influencing AI-2 Quorum sensing system, biofilm formation, and β-lactamase activity.

### 2.7 Kaempferol restores ceftiofur activity *in vivo*

**Figure 7:**
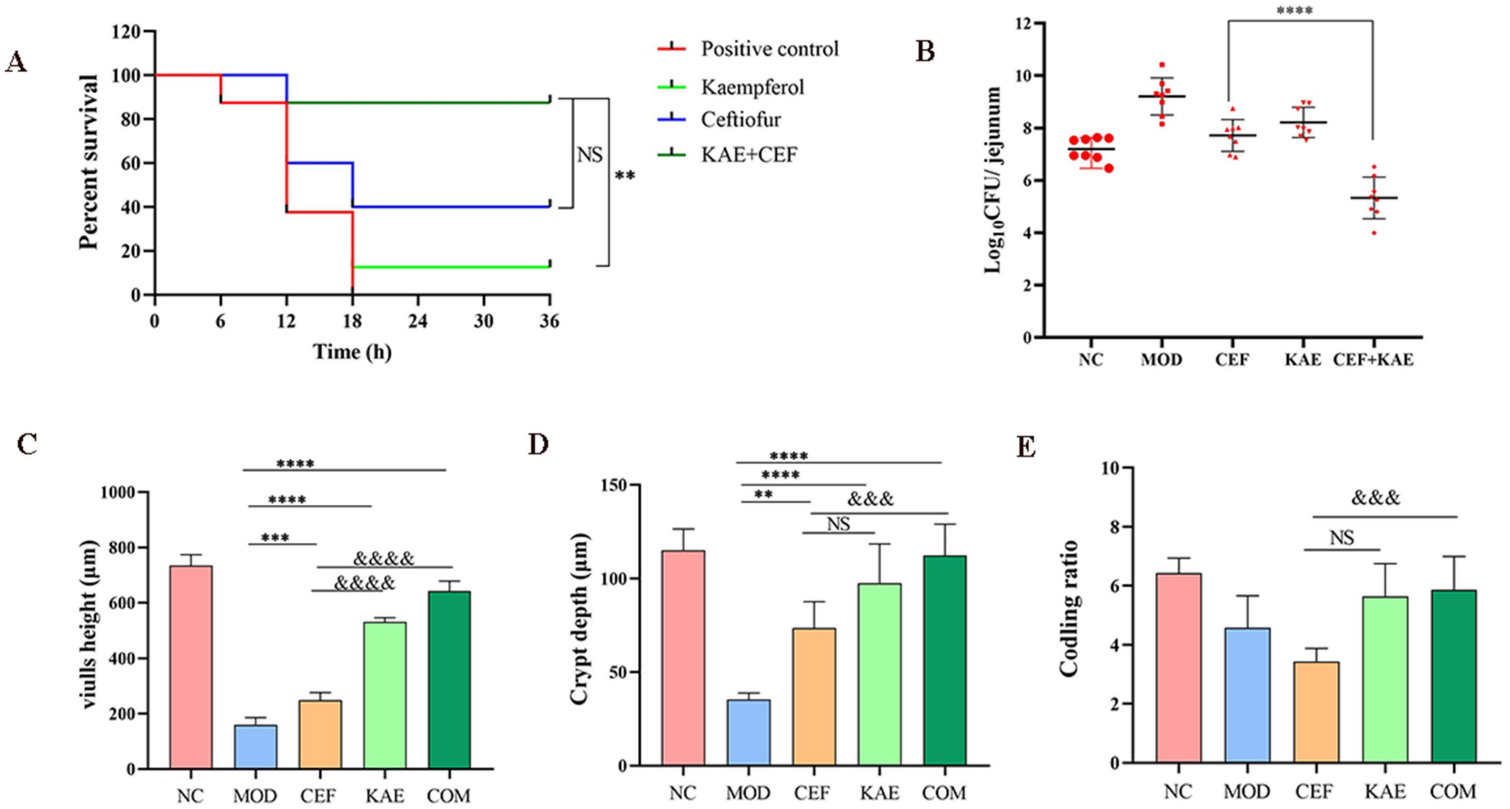
Kaempferol rescues ceftiofur activity *in vivo*. (A). Survival rates. (B). Intestinal bacterial load. (C). Length of villi in the small intestine. (D). Crypt depth. (E). Coding ratio. NC: Negative control; MOD: model group; CEF: ceftiofur group; KAE: kaempferol group; COM: combination group.

Considering the excellent synergistic bactericidal activity of the combination of Kaempferol and Ceftiofur against *E. coli in vitro*, we reasoned that kaempferol would reverse ceftiofur resistance *in vivo* and thus recover its clinical efficacy. To confirm this, a mouse intestinal inflammation model infected with *E. coli* (SY20) was constructed and used for this speculation. There was no significant difference between the COM group and the CEF group (*p* > 0.05), but the COM group obtained a survival benefit trend than the CEF group (Figure 7A). The COM group significantly reduced intestinal bacterial load than the CEF group (Figure 7B). Histopathology damage in mice with SY20 challenge was alleviated, as manifested by the higher villus height (Figure 7C) and crypt depth (Figure 7D) in the COM group and KAE group than in the CEF group, indicating that the integrity of intestinal villi was protected by kaempferol, but there was no significant difference in codling ratio between CEF group and KAE group (Figure 7E).

**Figure 8:**
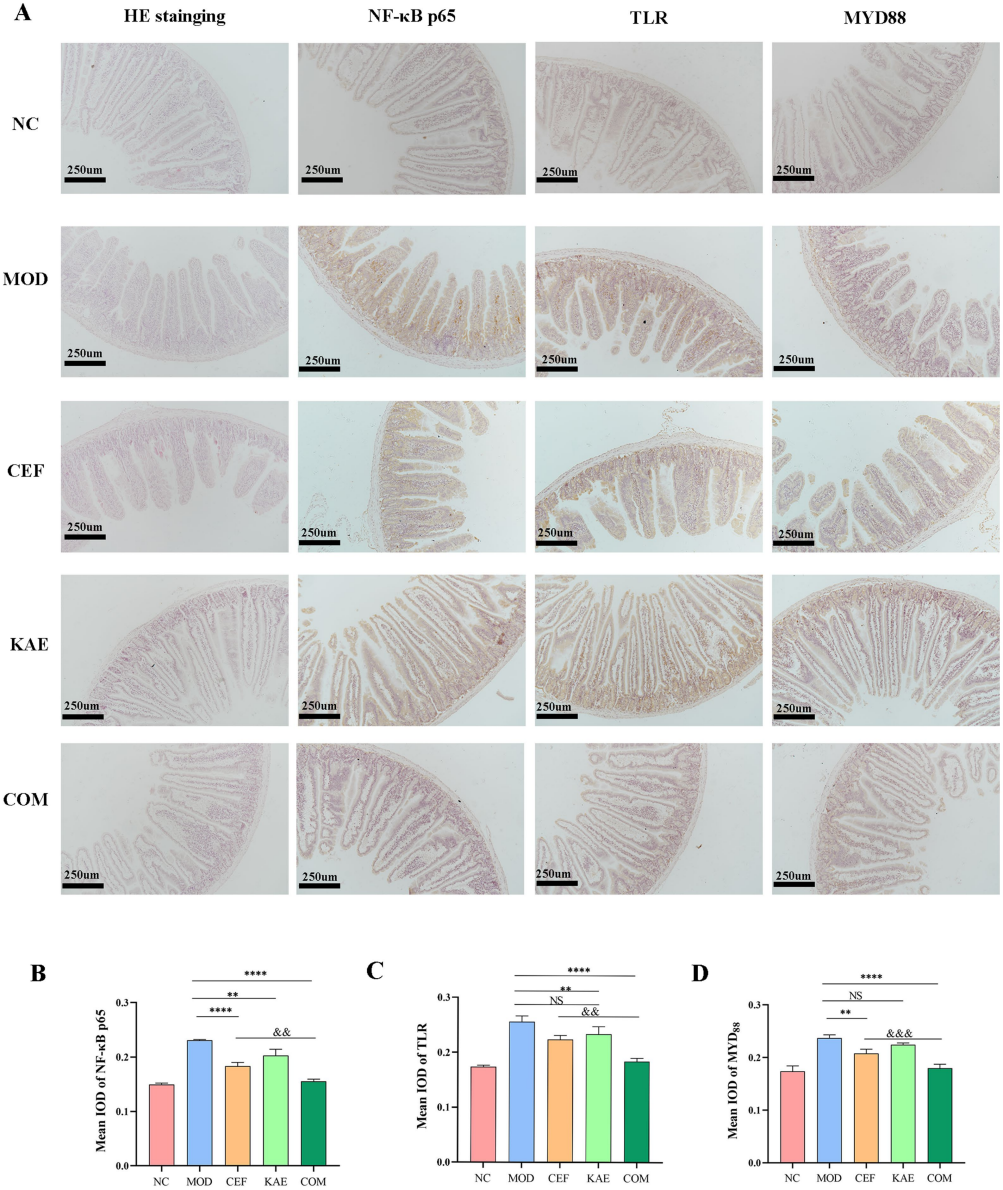
The distribution and expression analysis of inflammatory proteins and morphological changes in mice jejunum under different treatments. (A). HE staining and Immunohistochemistry. (B). The mean IOD of NF-κB. (C). The mean IOD of TLR. (D). The mean IOD of MYD_88._ NC:Negative control; MOD: model group; CEF: ceftiofur group; KAE: kaempferol group; COM: combination group.

As shown in Figure 8A, the jejunum villus was broken, and fragmentation occurred in the CEF group and MOD group. Unexpectedly, compared with the CEF group, the expression of NF-κB p65 proteins (Figure 8B), TLR proteins (Figure 8C), and MYD_88_ proteins (Figure 8D) were significantly reduced in the COM group. It is showed that kaempferol combined with ceftiofur exerted anti-inflammatory activity by affecting the NF-κB/TLR pathway (Figure 8A).

In conclusion, kaempferol fully restores the activity of ceftiofur in mous infection models by relieving TLR_4_/NF- κB pathway.

## 3 Discussion

In the prevailing epoch, antibiotic resistance is one of the critical threats (Hutchings et al., 2019). Despite the notion that ceftiofur has been widely recognized as one of the critical clinical antibiotics against *E. coli* infection, its clinical efficacy has greatly decreased due to the increasing resistance of *E. coli*. Therefore, the identification of potent adjuvants is of great importance (Wright, 2016).

As a previous study showed, kaempferol is a potential anti-inflammatory, antioxidant, and antibacterial compound (Chagas et al., 2022). Additionally, kaempferol can also inhibit the primary attachment phase of *Staphylococcus aureus* biofilm formation (Ming et al., 2017)^.^ In this study, kaempferol showed synergistic inhibitory against ESBLs *E. coli* combined with ceftiofur, which is the first report about the antibacterial effect of this combination against ESBLs *E. coli*.

The biofilm plays an important role in the formation of drug resistance in bacteria (Ciofu et al., 2022)^.^ Some previous studies found that mature biofilms can reduce the susceptibility of bacteria to ceftiofur (Ster et al., 2017). For example, the non-*Neisseria gonorrhoeae* suffered a lower survival rate than the aggregated *Neisseria gonorrhoeae* (Wang L C, et al., 2018)^,^ as the biofilm plays a key role in bacterial aggregation (Arnaouteli et al., 2021). In another previous study, kaempferol showed the ability to inhibit the initial attachment stage of *Staphylococcus aureus* biofilm formation by reducing the expression of adhesion-related genes (Chagas et al., 2022). As a result of that, we hypothesized that kaempferol could enhance the bacteriostatic effect of ceftiofur on *E. coli* by inhibiting the formation of biofilm. To study the possible mechanism of the antibacterial effect of kaempferol combined with ceftiofur, we evaluated the biofilm destruction activity of the combination. After being treated with kaempferol and ceftiofur together, the biofilm was significantly damaged, and its activity was inhibited. Furthermore, the effects of kaempferol on the adhesion and aggregation of *E. coli* in the initial stage were evaluated, the results showed found that the motility, adhesion, and self-aggregation of ESBLs *E. coli* were inhibited by the combination of ceftiofur and kaempferol, while kaempferol can increase the surface hydrophobicity of ESBLs *E. coli* when used alone, but the combination has no effect, this confirms our previous speculation. The effect of biofilm formation may be one of the reasons for the antibacterial effect of kaempferol combined with ceftiofur.

Quorum sensing (QS) system plays an important role in various bacterial processes, including drug resistance and biofilm formation, and it can help bacteria adapt to the external environment (Wang et al., 2023). AI-2 is a QS signal that mediates communication within and between many bacterial species (Wang et al., 2022)^,^ and the *LuxS* is a synthase involved in the synthesis of AI-2 (Wang Y, et al., 2018), inhibited the expression of the *LuxS* gene can affect the formation of biofilm in *E. coli* (Zong et al., 2024; Yu et al., 2021), the *LuxS* /AI-2 QS system of *E. coli* has been shown to regulate the formation of biofilms (Bai et al., 2022), it provides a new direction for inhibiting biofilms. Considering the anti-biofilm function of kaempferol, we speculate that kaempferol exerts its anti-biofilm effect by regulating the AI-2 QS system. So we measured the relative expression of *LuxS* /AI-2 system-related genes by RT-qPCR, including *luxS*, *LsrR*, and *LsrK*, *LsrR,* and *LsrK* regulate AI-2 uptake, playing a key role in the processing of AI-2, reducing the expression of LsrK and *LsrR* can inhibit the formation of AI-2, and then affect biofilm formation (Zuberi et al., 2017). Used Molecular Docking to confirm the interaction relationship between kaempferol and *LuxS* protein active center, and then we tested the effect of kaempferol on the molecular weight of the AI-2 signal. As expected, it is found that the kaempferol inhibited the AI-2 QS system and reduced the molecular weight of the AI-2 signal in ESBLs *E. coli*. Considering the anti-biofilm activity of kaempferol and its inhibition of *LuxS*/AI-2 QS, kaempferol might exert a synergistic effect on other antibiotics, more specific research needs to be conducted in the future.

Furthermore, ESBLs *E. coli* produce *β*-lactamases that hydrolyze β-lactam rings, thereby inactivating the drug, which is one of the main causes of resistance to β-lactams (Zhu et al., 2013; Nasrollahian et al., 2024). This study also pays attention to the effect of kaempferol on β-lactamases, and the isolates SY20 were *bla_CTX-M-27_* of β-lactamases used. The result shows that kaempferol can reduce the relative expression of the *bla_CTX-M-27_* gene, tightly bound to the active center of the *bla_CTX-M-27_* protein, inhibit the hydrolytic activity of *bla_CTX-M-27_* protein, this provides a new direction for our future research.

Previous studies (Qu et al., 2021) have shown that kaempferol can restore intestinal microbiota, and this study found that the bacterial load of the kaempferol group was lower than that of the ceftiofur group and the model group was also preliminarily verified. Besides, we found that kaempferol has a strong protective effect on intestinal villi, considering the role of villi in protecting the small intestine from bacterial invasion (Rostami Nejad et al., 2015), which may be one of the reasons for kaempferol affecting bacterial load. The anti-inflammatory of kaempferol has been partially reported. For example, kaempferol inhibits the activation of inflammatory NF-κB transcription factors through NIK/IKK and MAPKs in aged rat kidneys (Park et al., 2009), meanwhile, kaempferol alleviates enteritis in mice by inhibiting the kaempferol alleviates enteritis in mice by inhibiting the TLR4/NF-κB (Qu et al., 2021). Based on the aforesaid research, it was discovered that apart from the direct potentiation of ceftiofur, kaempferol assisted ceftiofur could alleviate inflammatory responses by modulating the NF-κB/TLR pathway through reducing the expression of NF-κB p65 proteins, TLR proteins, and MYD88 proteins. However, more works is still required to explain the underlying mechanisms of the anti-inflammatory and intestinal protective properties of kaempferol against ESBLs *E. coli* infections.

One potential limitation of the study is that the authors had to employ ESBLs *E. coli*. Although the study used ATCC®25922™ as the control to observe the combined antibacterial activity of kaempferol with ceftiofur against non-ESBLS *E.coli*, its inhibitory mechanism and *in vivo* therapeutic effect against non-ESBLS *E. coli* cannot be verified, this provides a new direction for our future work.

In conclusion, our data have shown that kaempferol exhibits potent synergistic activity with ceftiofur both *in vitro* and *in vivo*. The discovery of kaempferol as a novel ceftiofur adjuvant highlights the huge potential of compounds extracted from herbs against bacterial infections diseases. Nevertheless, the mechanism of this synergistic activity remains to be elucidated in the future.

## 4 Materials and methods

### 4.1 Strains

Strains proved to be ESBLs *E. coli* (Table 2) and were kept in the laboratory of the College of Veterinary Medicine, Northwest A&F University (Tong et al., 2023a).

**Table 2:** THE ESBLs gene information of isolates.

Isolates	ESBLs gene
SY13	*bla_CTX-M-14_*
SY20	*bla_CTX-M-27_*
SY22	*bla_CTX-M-27_*
YA1-3	*bla_TEM-1_*
YL2	*bla _CTX-M-9_*
YL6	*bla_IMP-4_*

### 4.2 Checkerboard assay

The combined antibacterial effect of kaempferol and ceftiofur was assessed by checkerboard assays (Tong et al., 2023a). In brief, both kaempferol and cefiofur were diluted to prepare seven gradient concentrations ranging from 1/16 MIC to 2 MIC. Each vertical column of tubes contained an identical concentration of drug A, while each horizontal row of tubes contained the same concentration of drug B. Bacterial suspension was inoculated into each tube to achieve a final density of approximately 1 × 10^6^ CFU/mL. Single-drug control tubes and blank control tubes were also prepared, with *E. coli* ATCC® 25,922™ used as a sensitivity control strain. Six ESBLs isolates were employed as experimental bacteria. All tubes were incubated at 37°C for 16 h under aerobic conditions. The experiment was conducted in triplicate. The fractional inhibitory concentration index (FICI) was calculated according to the following formula (Table 3).

**Table 3:** FICI values and criteria definitions.

FIC	Meaning
FICI ≤0.5	Synergistic effect
0.5 < FICI ≤0.75	Partial synergistic effect
0.75 < FICI ≤1	Additive effect
1 < FICI ≤4	Indifferent effect
FICI >4	Antagonism

FICI = MIC of kaempferol in combination/MIC of kaempferol alone + MIC of ceftiofur in combination/MIC of ceftiofur alone.

### 4.3 Time-kill curves

Time-kill assays (Liu et al., 2020) were employed to assess the synergistic antibacterial effects of kaempferol and ceftiofur against ESBL-producing *E. coli* by quantifying the reduction in CFU/mL over a 24-h period. Different concentrations of kaempferol and ceftiofur were co-incubated with an equal volume of ESBLS *E. coli* culture, while Mueller-Hinton broth (MHB) was used as a control in place of kaempferol or ceftiofur. All samples were incubated at 37°C, and aliquots (100 μL) were collected at 0, 2, 4, 6, 8, 10,12, and 24 h for colony counting after three rounds of centrifugation and resuspension to remove residual antimicrobial agents. Each assay was repeated in triplicate.

### 4.4 Growth curves

The growth curve was utilized to assess the inhibitory effect of the combination on ESBLs *E. coli* from the 24-h time point until the logarithmic phase. Kaempferol and ceftiofur were co-incubated with an equal volume of *E. coli* culture at 0.25 MIC of kaempferol and ceftiofur. MHB was added instead of kaempferol or ceftiofur as a control. The initial concentration of bacterial culture was 1 × 10^6^ CFU/mL. All samples were incubated at 37°C, and after 0, 2, 4, 6, 8, 12, and 24 h of incubation, 100 μL samples were extracted for measuring absorbance at OD_600._ Curves depicting absorbance changes at OD_600_ over time were plotted, with each assay being repeated in triplicate.

### 4.5 Resistance development studies

ESBLs *E. coli* in the exponential phase were diluted 1:1000 into fresh TSB media supplemented with 0.25 × MIC of ceftiofur or ceftiofur plus 0.25 × MIC of kaempferol. After incubation at 37°C for 24 h, the MIC of the culture was determined by two-fold serial dilutions in 96-well microtiter plates. Simultaneously, this culture was diluted to an adjusted 0.25 × MIC of drugs for subsequent passages. This process was repeated for a duration of 30 days, and the MIC values were measured at intervals of 0, 1, 2, 3, 6, 9, 12, 15, 18, 21, 24, 27, and 30 days. With each assay being repeated in triplicate.

### 4.6 Scanning electron microscope

The isolates were incubated in MHB with 0.25 MIC of kaempferol monotherapy, 0.25 MIC of ceftiofur monotherapy, or a combination of 0.25 MIC of kaempferol and 0.25 MIC of ceftiofur for 10 h. As a control, MHB was added instead of kaempferol or ceftiofur. After the incubation period, the bacteria were collected and washed with PBS, followed by fixation with 4% glutaraldehyde for 3 h. Subsequently, the bacteria underwent dehydration using graded ethanol before undergoing carbon dioxide critical point drying and gold spraying prior to scanning electron microscopy.

### 4.7 Crystal violet staining

The overnight bacterial cultures were adjusted to 0.5 McFarland in each well of 96-well microtiter plates using MHB broth. Subsequently, 10 μL of the diluted cultures were dispensed into each well and treated with kaempferol monotherapy, ceftiofur monotherapy, or a combination of kaempferol and ceftiofur. MHB was added to achieve a final volume of 200 μL/well. The microplates were completely covered with parafilm to prevent sample evaporation and incubated for 48 h. Planktonic cells were removed, and the attached cells were gently washed twice with a sterile physiological saline solution. Then, 200 μL of methanol/well was added and left for 20 min to fix the sessile cells. After discarding the methanol, the plates were left under a laminar flow cap until complete dryness (at least 30 min). Adhered cell staining was achieved by adding 200 μL of a 2% w/v crystal violet solution to each well for 20 min followed by gentle washing and drying. A volume of 200 μL of glacial acetic acid at a concentration of 20% w/v was added to release the bound dye (Caputo et al., 2022). The absorbance was measured at OD_570_. The concentration of all drugs mentioned above was set at 0.25 MIC level. Each assay was repeated in triplicate.

### 4.8 MTT staining

MTT staining was employed to evaluate the metabolic activity of biofilm cells. Overnight bacterial cultures, grown at the appropriate temperatures, were adjusted to 0.5 McFarland and then exposed to kaempferol monotherapy, ceftiofur monotherapy, or a combination of kaempferol and ceftiofur. After 48 h of incubation, the bacterial suspension was removed, and 150 μL of PBS and 30 μL of 0.3% MTT were added to microplates which were maintained at 37°C. Following a 2-h incubation period, the MTT solution was discarded; subsequently, after two washing steps with 200 μL of sterile physiological solution, 200 μL of DMSO was added for dissolution of formazan crystals before measuring absorbance at OD_595_. The concentration for all aforementioned drugs was set at 0.25 MIC. Triplicate tests were conducted, and average results were recorded for reproducibility.

### 4.9 Confocal laser scanning microscopy

As outlined in a previous investigation (Sun et al., 2021), biofilm formation in *E. coli* was evaluated using CLSM with slight adjustments. *E. coli* SY20 suspension supplemented with MHB was inoculated into a 24-well plate with a cover and incubated at 37°C for 24 h. Following the removal of the suspension, the wells were washed with PBS (pH = 7.2). After 4 h of treatment with kaempferol monotherapy, cefotifo monotherapy, or combined kaempferol and cefotifo, the solution was aspirated, and the wells were rinsed again with PBS (pH = 7.2). The biofilm was stained with PI and SYTO9 (PI stains dead bacteria, while SYTO9 stains all bacteria) for CLSM observation.

### 4.10 Swarming and swimming motility assays

As described in a previous study (De La Fuente-Núñez et al., 2012), swarming experiments were conducted on 1% LB agar plates for swarming and 0.3% LB agar plates for swimming, with the addition of four different treatments (negative control, 0.25MIC kaempferol, 0.25MIC ceftiofur, and 0.25MIC kaempferol +0.25MIC ceftiofur). *E. coli* drops were placed in the center of the agar plate, and the diameter was measured after a 24-h incubation period. Triplicate tests were conducted, and average results were recorded for reproducibility.

### 4.11 Fibrinogen-binding assay

In a previous study (Ming et al., 2017), the SY20 isolate was cultured overnight and then diluted 1:100 in sterile TSB. The culture was divided into four treatment groups, as detailed in section 4.10. Upon reaching an OD_600_ of 0.5, all cells were collected by centrifugation (5,000 × g for 5 min) and suspended in PBS to achieve an OD_600_ of 1.0. Subsequently, the resuspended cells were seeded onto polystyrene Costar 96-well plates coated with fibrinogen (pre-incubated overnight with 20 μg/mL bovine fibrinogen at 4°C) and incubated for 1 h at 37°C. After removing the supernatant, the cells were washed with PBS and fixed with a solution of 25% (v/v) formaldehyde for fixation. Following a duration of 30 min, the adherent bacteria underwent another round of washing with PBS before being stained with CV solution at a concentration of 12.5 g/L for a period of 10 min. The wells were subsequently washed again with PBS and allowed to dry before measuring different samples at OD_595._Triplicate tests were conducted, and average results were recorded for reproducibility.

### 4.12 Hydrophobicity and self-aggregation

The surface hydrophobicity of the SY20 isolate was investigated in four groups, as outlined in section 4.10. The bacterial culture was adjusted to a concentration of 1.0 × 10^5^ CFU/mL and subjected to four different treatments in TSB at 37°C for 4 h.

Cultured cells were centrifuged at 12,000× g at 4°C for 5 min. The resulting precipitates were then rinsed twice. The collected cells were re-dissolved in PBS to adjust the OD_600_ to 0.5 ± 0.05 (OD_initial_). Two milliliters of each suspension were mixed with chloroform (0.5 mL) and vortexed for 2 min. After incubating at room temperature for 15 min, the upper aqueous layer was collected, and its absorbance was measured at OD_600_ (OD_treatment_). The hydrophobicity (%) was calculated using the following equation (Ming et al., 2017):

Hydrophobicity (%) = (1 − OD_treatment_ / OD_initial_) × 100.

Auto-aggregation was performed using a modified version of the previously described method (Lee et al., 2021). The cell culture was adjusted to a final concentration of 1.0 × 10^5^ CFU/mL, and an *ε*-PL solution at 1/2 × MIC was mixed in a 1:1 ratio and then incubated at 37°C for 24 h. Non-treated cells were used as the control. Five milliliters of the mixture were collected and statically incubated at 4°C for 24 h. After incubation, the upper aqueous layer was measured at 600 nm (OD_treatment_). The sample was then vortexed and measured again at OD_600_ (OD_initial_). Auto-aggregation (%) was calculated using the following equation:

Auto-aggregation (%) = (1 − OD_treatment_/OD_initia_l) × 100.

### 4.13 AI-2 assays

To investigate the impact of kaempferol on AI-2 activity, SY20 isolates were cultured overnight at 37°C. The bacterial cultures were then diluted to a concentration of 10^5^ CFU/mL and divided into four test groups as outlined in section 4.10. Subsequently, the diluted bacterial cultures were incubated at 37°C for 12 h and centrifuged at 10,000 g for 10 min at 4°C. The negative control consisted of the supernatant obtained by centrifuging *E. coli* DH5α under the same culture conditions. The supernatants were filtered through a 0.22 μm filter and stored at −80°C. To assess AI-2 activity in each test group, V. harveyi BB170 cultured overnight at 28°C was diluted 5,000-fold with AB medium. Ninety microliters of the BB170 diluted culture, along with 10 μL of AI-2 supernatants (prepared from the above test group), were incubated at 28°C in the dark for 6 h, and the bioluminescence value was measured. The test was repeated 3 times independently. The test results are displayed in the form of ratio: luminescence value of each test group/luminescence value of *E. coli* DH5α (Li et al., 2021).

### 4.14 RT-qPCR

RT-qPCR was employed to assess the combined impact of kaempferol and ceftiofur on the LusX/AI-2 QS system-regulated genes in the SY20 The SY20 isolate was separately incubated in TSB containing kaempferol and ceftiofur, as well as only TSB, for 24 h. RNA extraction was performed using the triazole method, with RNA concentration determined by spectrophotometry and integrity assessed via agarose gel electrophoresis. Subsequently, cDNA synthesis was carried out using an Integrated First-strand cDNA Synthesis kit, followed by qRT-PCR analysis using 2 × Fast HS SYBR QPCR Mixture. The resulting qPCR data were analyzed for relative changes in gene expression levels based on the 2^−∆∆Ct^ method (Zuo et al., 2023). Primers listed in Table 4 were utilized for this study.

**Table 4:** Primer sequences used for qRT-PCR amplification.

Genes	Sequence (5′ to 3′)	Product size (bp)	Reference
16S rRNA-F	GCATAACGTCGCAAGACCAAAG	239	Wang Y, et al. (2018)
16S rRNA-R	TTCTTCATACACGCGGCATGG		
*Luxs*-F	GAAAACAATGAACACCCCGCATGG	92	Bai et al. (2022)
*Luxs*-R	TCCCTCTTTCTGGCATCACTTCTTTG		
*bla _ctx-m-27_-*F	GCTTTATGCGCAGACGAGTC	263	Design
*bla*_*CTX-M-2*7_-R	CATTGTGCCGTTGACGTGTT		
*LsrK*-F	GATGAACCTACCGCCTCGCTTAC	90	Bai et al. (2022)
*LsrK*-R	AACAATACCCACGCCAGTAGCAAG		
*LsrR*-F	AACAATACCCACGCCAGTAGCAAG	143	Bai et al. (2022)
*LsrR*-R	GCTGCCCGATTCCCGTCATATAAG		

### 4.15 Molecular docking assay

To assess the binding affinity of kaempferol with *LuxS* protein and *bla_CTX-M-27_* protein, we conducted a molecular docking assay using Autodock4 software. The chemical structure of kaempferol was retrieved from the PubChem database, while the structures of *E. coli LuxS* protein and *bla_CTX-M-27_* protein were obtained from UniProtKB. We assessed the binding capability between kaempferol and *LuxS* protein using Autodock Vina, Pymol was used to visualize the results (Huang, 2023). Furthermore, binding scores lower than −5 Kcal/mol were indicative of binding activity.

### 4.16 Nitrocefin assay

Nitrocefin assay (Teng et al., 2019) was used to assess the effect of kaempferol on *bla_CTX-M-27_* protein activity. The SY20 isolate was cultured to OD600 nm = 0.6 at 37°C, after centrifugation, the bacteria were resuspended in sterile phosphate buffer (pH = 7.2) and broken by ultrasound in an ice bath. After the completion of the ultrasound, *bla_CTX-M-27_* protein crude extract was obtained from the supernatant after centrifugation at 12000 rpm for 10 min at 4°C. *Bla_CTX-M-27_* protein rude extract was incubated with various concentrations of kaempferol (128, 256, 512 μg/mL) in phosphate buffer (pH = 7.2) at 37°C for 5 min, and then, 50 μg/mL of nitrocefin was added to the mixture. After 10 min of incubation, the samples were read at OD_492_ nm to determine the level of nitrocefin hydrolysis.

### 4.17 Transcriptome analysis

Transcriptome sequencing services were conducted by Novogene-Beijing. SY20 of ESBLs E.coli were treated 0.25MIC kaempferol and 0.25MIC kaempferol +0.25MIC ceftiofur procedure involved reactivating the test bacteria in 400 mL of MH broth and incubating them at 37°C for 4 h until reaching the early log phase. Subsequently, drug solutions were added to achieve the desired final concentration and then incubated at 37°C for an additional 4 h. The cultures were then centrifuged at 4°C, the supernatant was discarded, and the samples were flash-frozen in liquid nitrogen. Utilizing established RNA extraction protocols. RNA integrity and total quantity were evaluated using an Agilent 2,100 bioanalyzer. Ribosomal RNA (rRNA) was depleted from the total RNA to enrich for mRNA using probes, and libraries were constructed in a strand-specific manner. Subsequently, different libraries were pooled based on effective concentration and desired sequencing output for Illumina sequencing, which involved quality analysis of the sequencing, quantitative gene expression analysis, GO enrichment analysis, KEGG enrichment analysis, GSEA enrichment analysis, etc.

### 4.18 Mouse jejunum inflammation model

All mice were divided into five groups, with eight female SPF Kunming mice in each group receiving intraperitoneal injections of the lowest lethal dose of *E. coli* SY20 suspension at a concentration of 1 × 10^8^ CFU/mL. Following a 2-h infection period, the mice were administered a single intraperitoneal dose of kaempferol (50 mg/kg, KAE group), ceftiofur (50 mg/kg, CEF group), or kaempferol and ceftiofur (50 + 50 mg/kg, COM group). The negative control group received 0.9% Nacl injections (NC group). The survival rate of the treated mice was monitored for up to 36 h.

### 4.19 HE staining and immunohistochemistry

The jejunum were collected, washed with PBS, fixed with a 4% paraformaldehyde solution, dehydrated with ethanol, embedded in paraffin, sliced, and stained with Hematoxylin & Eosin (HE) staining. The samples were examined with a microscope, and the villi length and crypt depth were recorded (Tang et al., 2021).

After fixation in 4% paraformaldehyde solution, the jejunum sections were embedded and subjected to immunohistochemical staining for detection of TLR4, NF-κB p65, and MYD88 antibodies. The samples were observed by staining with hematoxylin. Finally, the samples were examined with a microscope, and the results were measured. Three fields were observed in each sample,and the integrated optical density (IOD) was calculated (Tang et al., 2021).

### 4.20 Statistical analysis

Statistical analysis was conducted using GraphPad Prism 8 and SPSS software. The data are presented as the mean ± SD. The statistical significance of differences was assessed using a t-test for two groups or a one-way ANOVA test for multiple groups, the *p*-value of survival rates were determined by log-rank test. *p* value <0.05 was considered statistically significant for all comparisons. All figures were generated using GraphPad Prism 8.0.1 and edited with Photoshop 2021.

## Data Availability

The original contributions presented in the study are publicly available. This data can be found here: [https://doi.org/10.6084/m9.figshare.27686070.v1].

## References

[ref1] Aleksandrowicz A. Carolak E. Dutkiewicz A. Błachut A. Waszczuk W. Grzymajlo K. (2023). Better together-Salmonella biofilm-associated antibiotic resistance. Gut Microbes 15:2229937. doi: 10.1080/19490976.2023.2229937, PMID: 37401756 PMC10321201

[ref2] Arnaouteli S. Bamford N. C. Stanley-Wall N. R. Kovács Á. T. (2021). *Bacillus subtilis* biofilm formation and social interactions. Nat. Rev. Microbiol. 19, 600–614. doi: 10.1038/s41579-021-00540-9, PMID: 33824496

[ref3] Bai Y. B. Shi M. Y. Wang W. W. Wu L. Y. Bai Y. T. Li B.. (2022). Novel quorum sensing inhibitor Echinatin as an antibacterial synergist against *Escherichia coli*. Front. Microbiol. 13:1003692. doi: 10.3389/fmicb.2022.1003692, PMID: 36386683 PMC9663819

[ref4] Caputo L. Capozzolo F. Amato G. De Feo V. Fratianni F. Vivenzio G.. (2022). Chemical composition, antibiofilm, cytotoxic, and anti-acetylcholinesterase activities of *Myrtus communis* L. leaves essential oil. BMC Complement Med Ther. 22:142. doi: 10.1186/s12906-022-03583-4, PMID: 35596201 PMC9123742

[ref5] Chagas M. D. S. S. Behrens M. D. Moragas-Tellis C. J. Penedo G. X. M. Silva A. R. Gonçalves-de-Albuquerque C. F. (2022). Flavonols and flavones as potential anti-inflammatory, antioxidant, and antibacterial compounds. Oxidative Med. Cell. Longev. 2022, 1–21. doi: 10.1155/2022/9966750, PMID: 36111166 PMC9470311

[ref6] Chang S. Li X. Zheng Y. Shi H. Zhang D. Jing B.. (2022). Kaempferol exerts a neuroprotective effect to reduce neuropathic pain through TLR4/NF-ĸB signaling pathway. Phytother. Res. 36, 1678–1691. doi: 10.1002/ptr.7396, PMID: 35234314 PMC9311149

[ref7] Ciofu O. Moser C. Jensen P. Ø. Høiby N. (2022). Tolerance and resistance of microbial biofilms. Nat. Rev. Microbiol. 20, 621–635. doi: 10.1038/s41579-022-00682-4 35115704

[ref8] de la Fuente-Núñez C. Korolik V. Bains M. Nguyen U. Breidenstein E. B. Horsman S.. (2012). Inhibition of bacterial biofilm formation and swarming motility by a small synthetic cationic peptide. Antimicrob. Agents Chemother. 56, 2696–2704. doi: 10.1128/AAC.00064-12, PMID: 22354291 PMC3346644

[ref9] Huang S. (2023). Efficient analysis of toxicity and mechanisms of environmental pollutants with network toxicology and molecular docking strategy: acetyl tributyl citrate as an example. Sci. Total Environ. 905:167904. doi: 10.1016/j.scitotenv.2023.167904, PMID: 37858827

[ref10] Hutchings M. I. Truman A. W. Wilkinson B. (2019). Antibiotics: past, present and future. Curr. Opin. Microbiol. 51, 72–80. doi: 10.1016/j.mib.2019.10.008 31733401

[ref11] Lee D. U. Park Y. J. Yu H. H. Jung S. C. Park J. H. Lee D. H.. (2021). Antimicrobial and Antibiofilm effect of ε-Polylysine against *Salmonella Enteritidis*, *listeria monocytogenes*, and *Escherichia coli* in tryptic soy broth and chicken juice. Food Secur. 10:2211. doi: 10.3390/foods10092211, PMID: 34574320 PMC8466587

[ref12] Li J. Fan Q. Jin M. Mao C. Zhang H. Zhang X.. (2021). Paeoniflorin reduce *luxS*/AI-2 system-controlled biofilm formation and virulence in *Streptococcus suis*. Virulence 12, 3062–3073. doi: 10.1080/21505594.2021.2010398, PMID: 34923916 PMC8923065

[ref13] Lin S. Li H. Tao Y. Liu J. Yuan W. Chen Y.. (2020). In vitro and in vivo evaluation of membrane-active flavone Amphiphiles: semisynthetic Kaempferol-derived antimicrobials against drug-resistant gram-positive Bacteria. J. Med. Chem. 63, 5797–5815. doi: 10.1021/acs.jmedchem.0c00053, PMID: 32400157

[ref14] Lin Y. Zhang Y. Liu S. Ye D. Chen L. Huang N.. (2021). Quercetin rejuvenates sensitization of Colistin-resistant *Escherichia coli* and *Klebsiella Pneumoniae* clinical isolates to Colistin. Front. Chem. 9:795150. doi: 10.3389/fchem.2021.795150, PMID: 34900948 PMC8656154

[ref15] Liu Y. Jia Y. Yang K. Li R. Xiao X. Zhu K.. (2020). Metformin restores Tetracyclines susceptibility against multidrug resistant Bacteria. Adv Sci (Weinh). 7:1902227. doi: 10.1002/advs.201902227, PMID: 32596101 PMC7312304

[ref16] Ming D. Wang D. Cao F. Xiang H. Mu D. Cao J.. (2017). Kaempferol inhibits the primary attachment phase of biofilm formation in *Staphylococcus aureus*. Front. Microbiol. 8:2263. doi: 10.3389/fmicb.2017.02263, PMID: 29187848 PMC5694784

[ref17] Muñoz K. A. Ulrich R. J. Vasan A. K. Sinclair M. Wen P. C. Holmes J. R.. (2024). A gram-negative-selective antibiotic that spares the gut microbiome. Nature 630, 429–436. doi: 10.1038/s41586-024-07502-0, PMID: 38811738 PMC12135874

[ref18] Nasrollahian S. Graham J. P. Halaji M. (2024). A review of the mechanisms that confer antibiotic resistance in pathotypes of *E. coli*. Front. Cell. Infect. Microbiol. 14:1387497. doi: 10.3389/fcimb.2024.1387497, PMID: 38638826 PMC11024256

[ref19] Park M. J. Lee E. K. Heo H. S. Kim M. S. Sung B. Kim M. K.. (2009). The anti-inflammatory effect of kaempferol in aged kidney tissues: the involvement of nuclear factor-kappaB via nuclear factor-inducing kinase/IkappaB kinase and mitogen-activated protein kinase pathways. J. Med. Food 12, 351–358. doi: 10.1089/jmf.2008.0006, PMID: 19459737 PMC6469524

[ref20] Periferakis A. Periferakis K. Badarau I. A. Petran E. M. Popa D. C. Caruntu A.. (2022). Kaempferol: antimicrobial properties, sources, clinical, and traditional applications. Int. J. Mol. Sci. 23:15054. doi: 10.3390/ijms232315054, PMID: 36499380 PMC9740324

[ref21] Porras G. Chassagne F. Lyles J. T. Marquez L. Dettweiler M. Salam A. M.. (2021). Ethnobotany and the role of plant natural products in antibiotic drug discovery. Chem. Rev. 121, 3495–3560. doi: 10.1021/acs.chemrev.0c00922, PMID: 33164487 PMC8183567

[ref22] Qu Y. Li X. Xu F. Zhao S. Wu X. Wang Y.. (2021). Kaempferol alleviates murine experimental colitis by restoring gut microbiota and inhibiting the LPS-TLR4-NF-κB Axis. Front. Immunol. 12:679897. doi: 10.3389/fimmu.2021.679897, PMID: 34367139 PMC8339999

[ref23] Rostami Nejad M. Ishaq S. Al Dulaimi D. Zali M. R. Rostami K. (2015). The role of infectious mediators and gut microbiome in the pathogenesis of celiac disease. Arch. Iran. Med. 18, 244–249., PMID: 25841946

[ref24] Ster C. Lebeau V. Leclerc J. Fugère A. Veh K. A. Roy J. P.. (2017). In vitro antibiotic susceptibility and biofilm production of *Staphylococcus aureus* isolates recovered from bovine intramammary infections that persisted or not following extended therapies with cephapirin, pirlimycin or ceftiofur. Vet. Res. 48:56. doi: 10.1186/s13567-017-0463-0, PMID: 28934980 PMC5609010

[ref25] Sun Y. Jiang W. Zhang M. Zhang L. Shen Y. Huang S.. (2021). The inhibitory effects of Ficin on *Streptococcus mutans* biofilm formation. Biomed. Res. Int. 2021, 1–11. doi: 10.1155/2021/6692328, PMID: 33860052 PMC8009705

[ref26] Tang J. Xu L. Zeng Y. Gong F. (2021). Effect of gut microbiota on LPS-induced acute lung injury by regulating the TLR4/NF-kB signaling pathway. Int. Immunopharmacol. 91:107272. doi: 10.1016/j.intimp.2020.107272, PMID: 33360370

[ref27] Teng Z. Guo Y. Liu X. Zhang J. Niu X. Yu Q.. (2019). Theaflavin-3, 3′-digallate increases the antibacterial activity of β-lactam antibiotics by inhibiting metallo-β-lactamase activity. J. Cell. Mol. Med. 23, 6955–6964. doi: 10.1111/jcmm.14580, PMID: 31392792 PMC6787515

[ref28] Tong Y. C. Li P. C. Yang Y. Lin Q. Y. Liu J. T. Gao Y. N.. (2023a). Detection of antibiotic resistance in feline-origin ESBL *Escherichia coli* from different areas of China and the resistance elimination of garlic oil to Cefquinome on ESBL *E. coli*. Int. J. Mol. Sci. 24:9627. doi: 10.3390/ijms24119627, PMID: 37298578 PMC10253432

[ref29] Tong Y. C. Zhang Y. N. Li P. C. Cao Y. L. Ding D. Z. Yang Y.. (2023b). Detection of antibiotic-resistant canine origin *Escherichia coli* and the synergistic effect of magnolol in reducing the resistance of multidrug-resistant *Escherichia coli*. Front Vet Sci. 10:1104812. doi: 10.3389/fvets.2023.1104812, PMID: 37008355 PMC10057116

[ref30] Wang Y. Bian Z. Wang Y. (2022). Biofilm formation and inhibition mediated by bacterial quorum sensing. Appl. Microbiol. Biotechnol. 106, 6365–6381. doi: 10.1007/s00253-022-12150-3 36089638

[ref31] Wang M. Lian Y. Wang Y. Zhu L. (2023). The role and mechanism of quorum sensing on environmental antimicrobial resistance. Environ. Pollut. 322:121238. doi: 10.1016/j.envpol.2023.121238 36758922

[ref32] Wang L. C. Litwin M. Sahiholnasab Z. Song W. Stein D. C. (2018). *Neisseria gonorrhoeae* aggregation reduces its ceftriaxone susceptibility. Antibiotics (Basel). 7:48. doi: 10.3390/antibiotics7020048, PMID: 29914058 PMC6022932

[ref33] Wang Y. Wang Y. Sun L. Grenier D. Yi L. (2018). The LuxS/AI-2 system of *Streptococcus suis*. Appl. Microbiol. Biotechnol. 102, 7231–7238. doi: 10.1007/s00253-018-9170-7, PMID: 29938319

[ref34] Wei S. Yang Y. Tian W. Liu M. Yin S. Li J. (2020). Synergistic activity of fluoroquinolones combining with Artesunate against multidrug-resistant *Escherichia coli*. Microb. Drug Resist. 26, 81–88. doi: 10.1089/mdr.2018.0463, PMID: 31738637 PMC6978754

[ref35] Wright G. D. (2016). Antibiotic adjuvants: rescuing antibiotics from resistance. Trends Microbiol. 24, 862–871. doi: 10.1016/j.tim.2016.06.009 27430191

[ref36] Yang Y. Chen Z. Zhao X. Xie H. Du L. Gao H.. (2022). Mechanisms of Kaempferol in the treatment of diabetes: a comprehensive and latest review. Front Endocrinol (Lausanne). 13:990299. doi: 10.3389/fendo.2022.990299, PMID: 36157449 PMC9490412

[ref37] Yu T. Ma M. Sun Y. Xu X. Qiu S. Yin J.. (2021). The effect of sublethal concentrations of benzalkonium chloride on the LuxS/AI-2 quorum sensing system, biofilm formation and motility of *Escherichia coli*. Int. J. Food Microbiol. 353:109313. doi: 10.1016/j.ijfoodmicro.2021.109313, PMID: 34175578

[ref38] Zhu J. Hixon M. S. Globisch D. Kaufmann G. F. Janda K. D. (2013). Mechanistic insights into the LsrK kinase required for autoinducer-2 quorum sensing activation. J. Am. Chem. Soc. 135, 7827–7830. doi: 10.1021/ja4024989, PMID: 23672516 PMC3736694

[ref39] Zong B. Xiao Y. Wang P. Liu W. Ren M. Li C.. (2024). Baicalin weakens the virulence of porcine Extraintestinal pathogenic *Escherichia coli* by inhibiting the LuxS/AI-2 quorum-sensing system. Biomol. Ther. 14:452. doi: 10.3390/biom14040452, PMID: 38672469 PMC11047829

[ref40] Zuberi A. Misba L. Khan A. U. (2017). CRISPR interference (CRISPRi) inhibition of luxS gene expression in *E. coli*: an approach to inhibit biofilm. Front. Cell. Infect. Microbiol. 7:214. doi: 10.3389/fcimb.2017.00214, PMID: 28603699 PMC5445563

[ref41] Zuo J. Shen Y. Wang H. Gao S. Yuan S. Song D.. (2023). Effects of metformin on *Streptococcus suis* LuxS/AI-2 quorum sensing system and biofilm formation. Microb. Pathog. 181:106183. doi: 10.1016/j.micpath.2023.106183, PMID: 37263449

